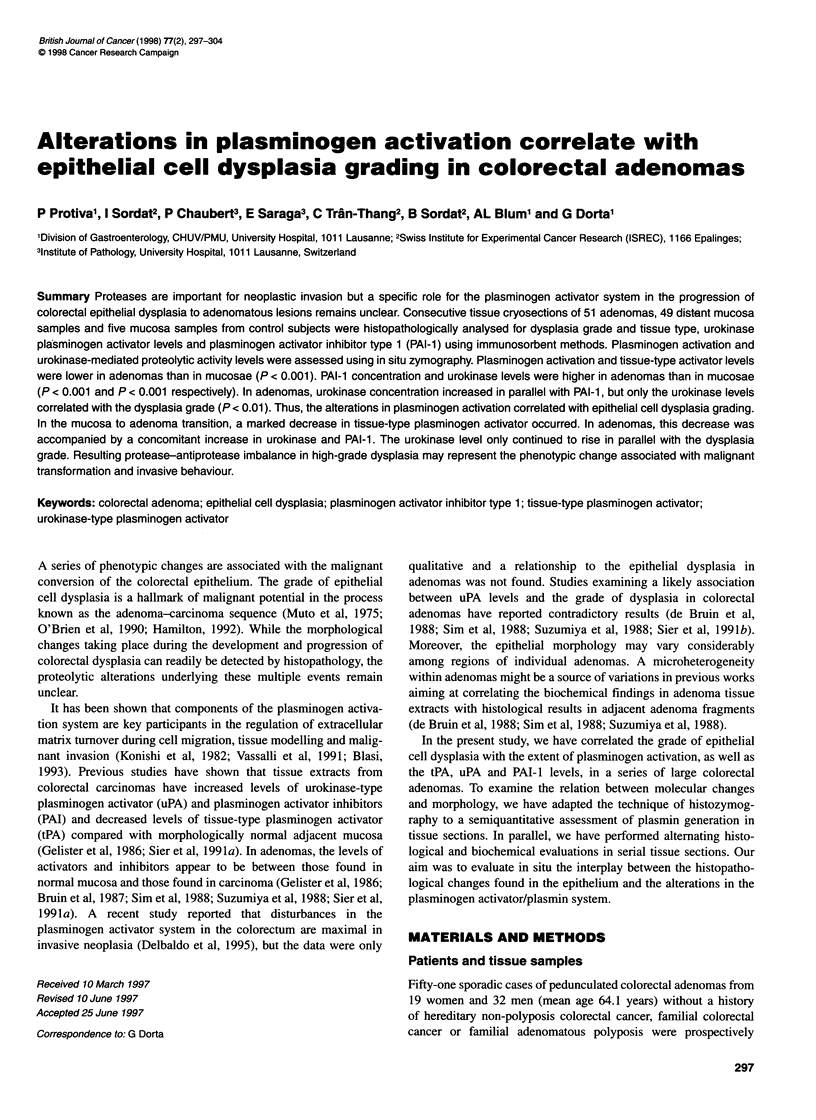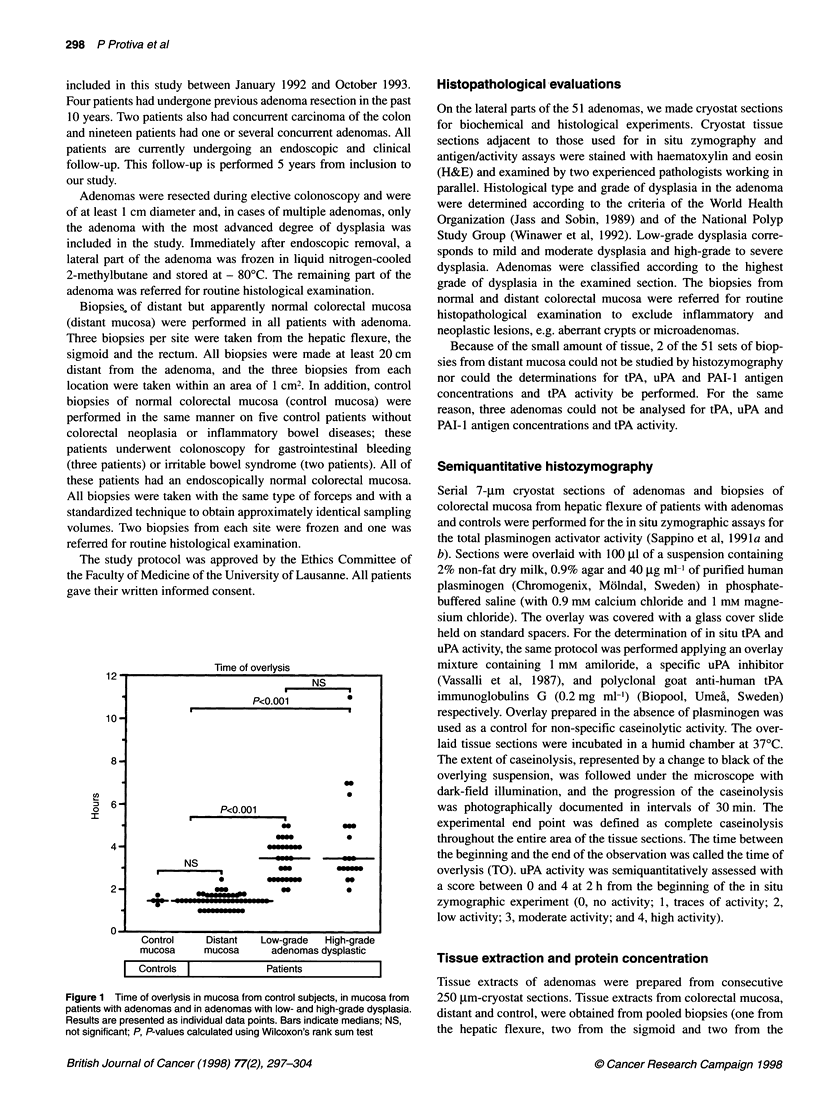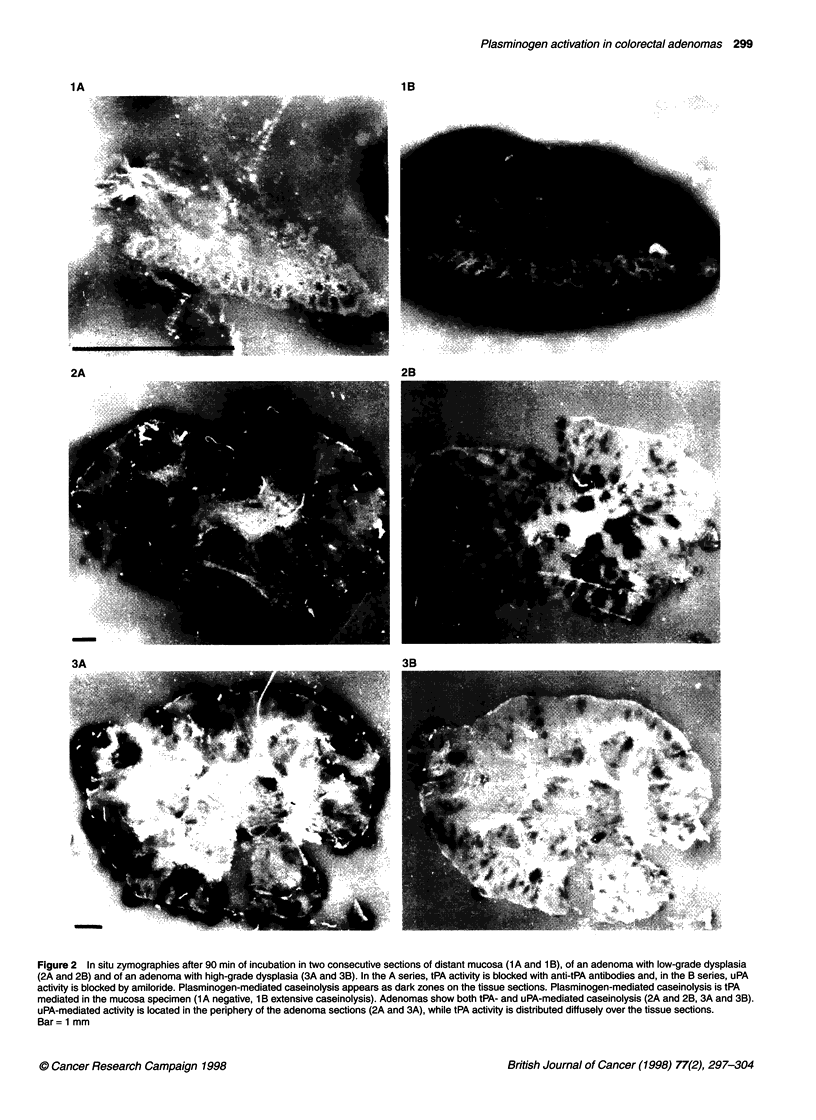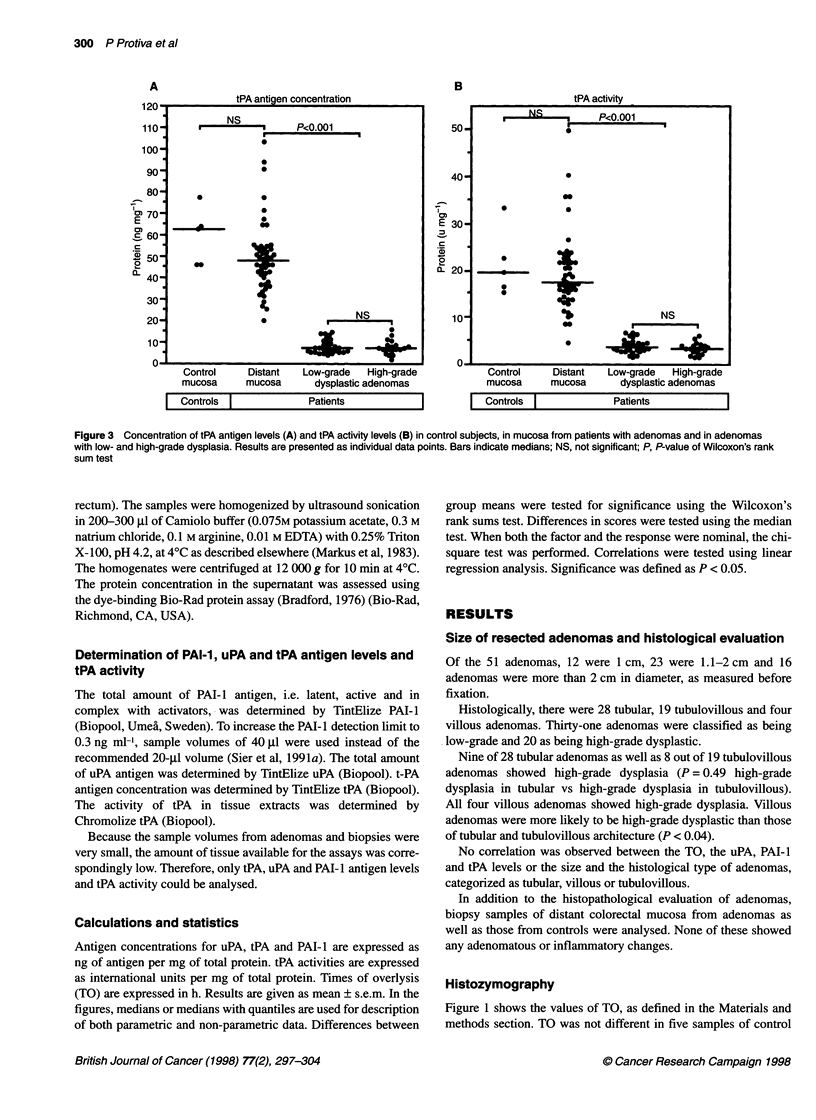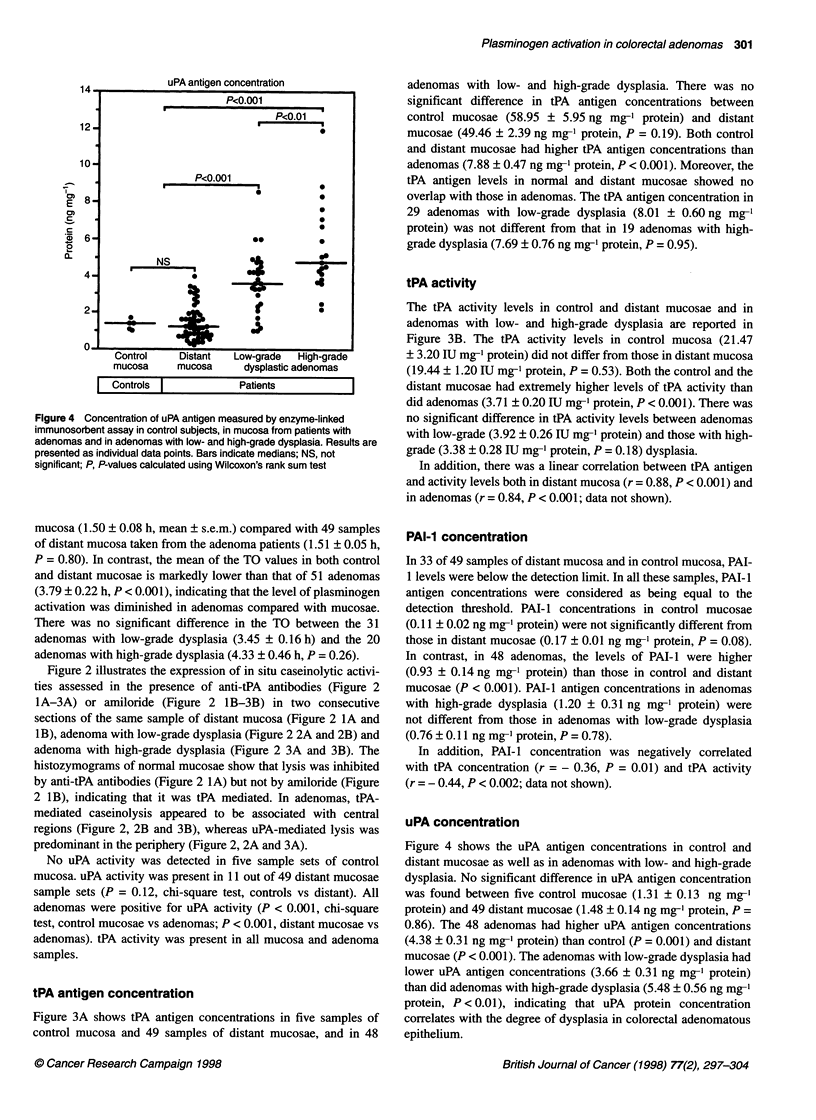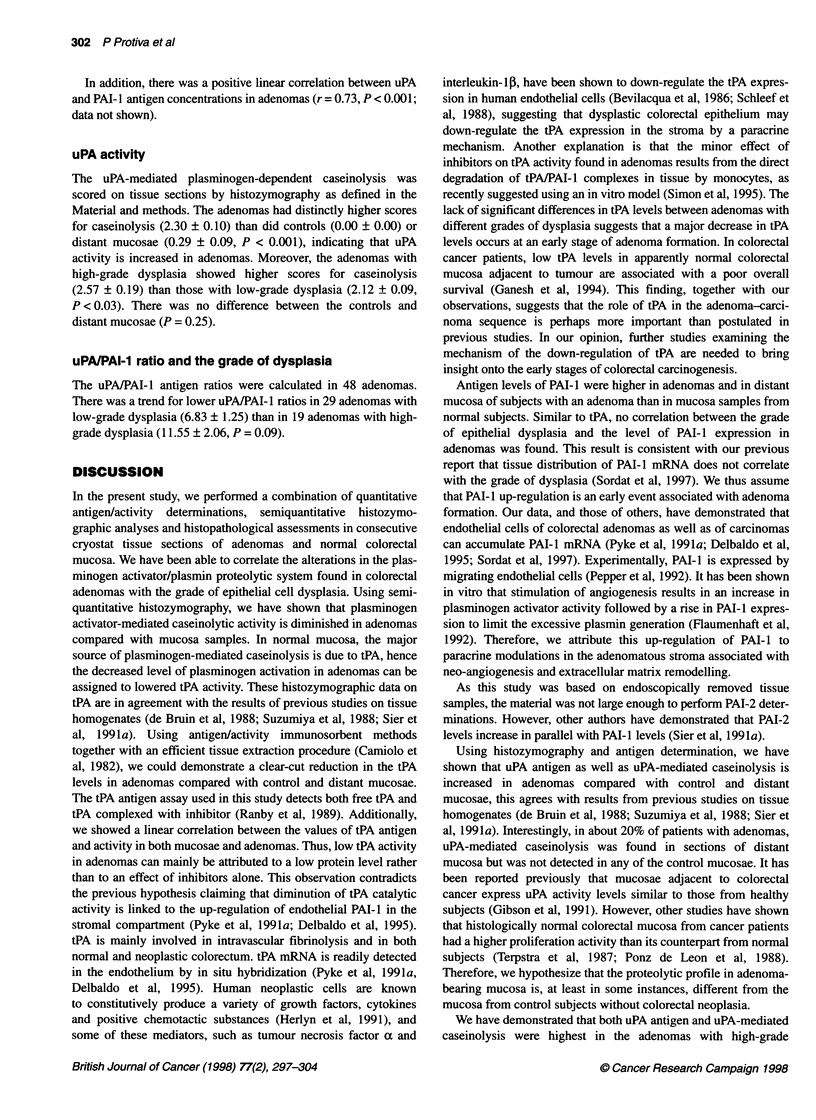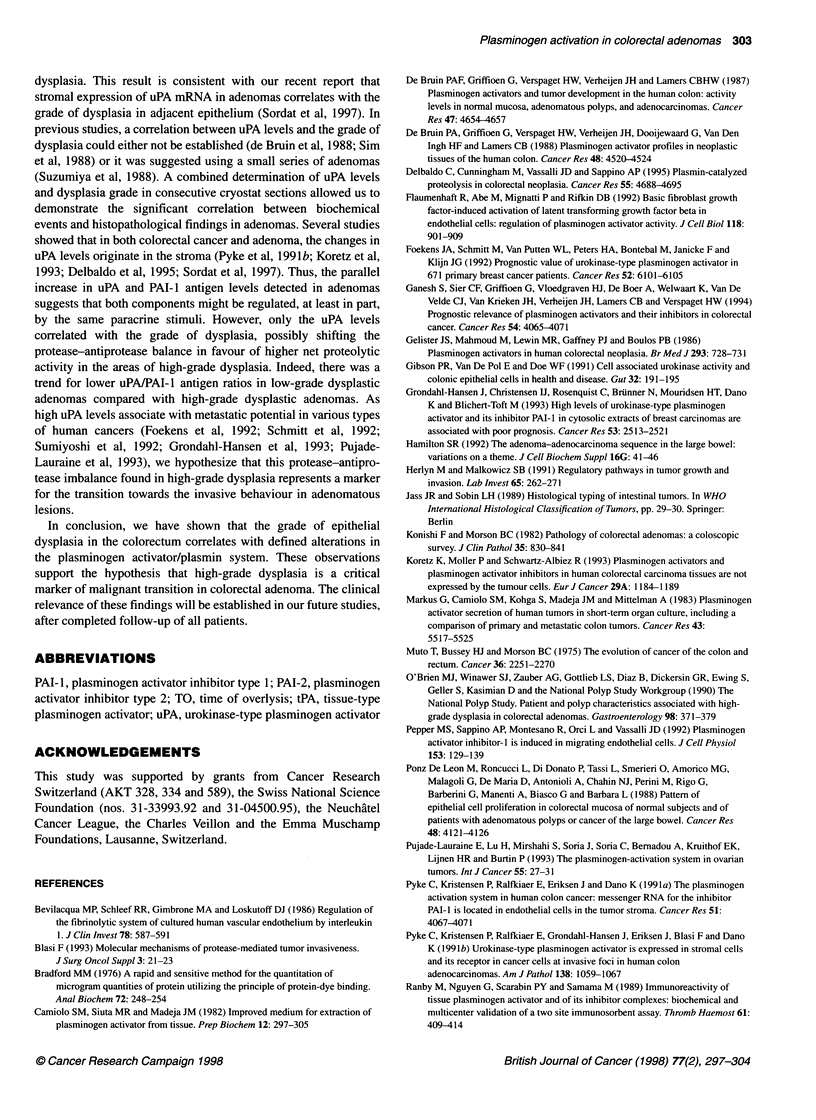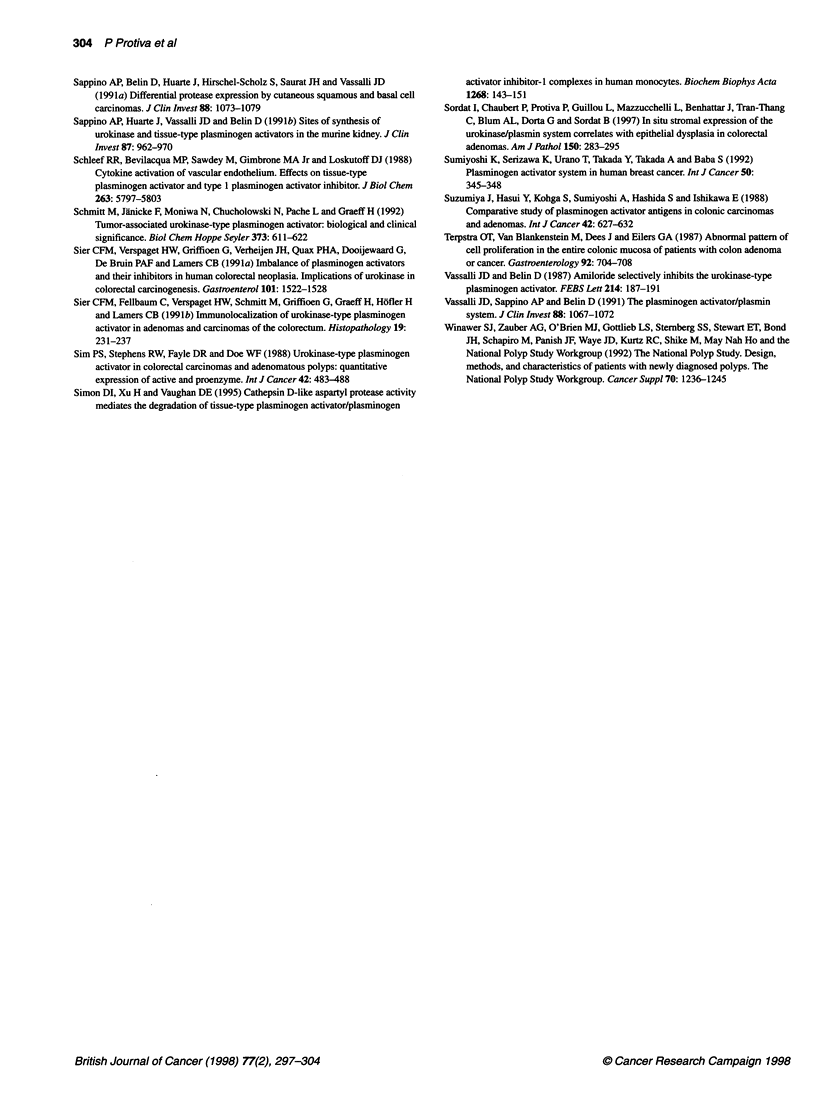# Alterations in plasminogen activation correlate with epithelial cell dysplasia grading in colorectal adenomas.

**DOI:** 10.1038/bjc.1998.46

**Published:** 1998

**Authors:** P. Protiva, I. Sordat, P. Chaubert, E. Saraga, C. TrÃ¢n-Thang, B. Sordat, A. L. Blum, G. Dorta

**Affiliations:** Division of Gastroenterology, CHUV/PMU, University Hospital, Lausanne, Switzerland.

## Abstract

**Images:**


British Journal of Cancer (1998) 77(2), 297–304
© 1998 Cancer Research Campaign

### Alterations in plasminogen activation correlate with epithelial cell dysplasia grading in colorectal adenomas

**P Protiva[1], I Sordat[2], P Chaubert[3], E Saraga[3], C Trân-Thang[2], B Sordat[2], AL Blum[1] and G Dorta[1]**

[1]Division of Gastroenterology, CHUV/PMU, University Hospital, 1011 Lausanne; [2]Swiss Institute for Experimental Cancer Research (ISREC), 1166 Epalinges; [3]Institute of Pathology, University Hospital, 1011 Lausanne, Switzerland

**Summary** Proteases are important for neoplastic invasion but a specific role for the plasminogen activator system in the progression of colorectal epithelial dysplasia to adenomatous lesions remains unclear. Consecutive tissue cryosections of 51 adenomas, 49 distant mucosa samples and five mucosa samples from control subjects were histopathologically analysed for dysplasia grade and tissue type, urokinase plasminogen activator levels and plasminogen activator inhibitor type 1 (PAI-1) using immunosorbent methods. Plasminogen activation and urokinase-mediated proteolytic activity levels were assessed using in situ zymography. Plasminogen activation and tissue-type activator levels were lower in adenomas than in mucosae ($P < 0.001$). PAI-1 concentration and urokinase levels were higher in adenomas than in mucosae ($P < 0.001$ and $P < 0.001$ respectively). In adenomas, urokinase concentration increased in parallel with PAI-1, but only the urokinase levels correlated with the dysplasia grade ($P < 0.01$). Thus, the alterations in plasminogen activation correlated with epithelial cell dysplasia grading. In the mucosa to adenoma transition, a marked decrease in tissue-type plasminogen activator occurred. In adenomas, this decrease was accompanied by a concomitant increase in urokinase and PAI-1. The urokinase level only continued to rise in parallel with the dysplasia grade. Resulting protease–antiprotease imbalance in high-grade dysplasia may represent the phenotypic change associated with malignant transformation and invasive behaviour.

**Keywords:** colorectal adenoma; epithelial cell dysplasia; plasminogen activator inhibitor type 1; tissue-type plasminogen activator; urokinase-type plasminogen activator

A series of phenotypic changes are associated with the malignant conversion of the colorectal epithelium. The grade of epithelial cell dysplasia is a hallmark of malignant potential in the process known as the adenoma–carcinoma sequence (Muto et al, 1975; O'Brien et al, 1990; Hamilton, 1992). While the morphological changes taking place during the development and progression of colorectal dysplasia can readily be detected by histopathology, the proteolytic alterations underlying these multiple events remain unclear.

It has been shown that components of the plasminogen activation system are key participants in the regulation of extracellular matrix turnover during cell migration, tissue modelling and malignant invasion (Konishi et al, 1982; Vassalli et al, 1991; Blasi, 1993). Previous studies have shown that tissue extracts from colorectal carcinomas have increased levels of urokinase-type plasminogen activator (uPA) and plasminogen activator inhibitors (PAI) and decreased levels of tissue-type plasminogen activator (tPA) compared with morphologically normal adjacent mucosa (Gelister et al, 1986; Sier et al, 1991*a*). In adenomas, the levels of activators and inhibitors appear to be between those found in normal mucosa and those found in carcinoma (Gelister et al, 1986; Bruin et al, 1987; Sim et al, 1988; Suzumiya et al, 1988; Sier et al, 1991*a*). A recent study reported that disturbances in the plasminogen activator system in the colorectum are maximal in invasive neoplasia (Delbaldo et al, 1995), but the data were only qualitative and a relationship to the epithelial dysplasia in adenomas was not found. Studies examining a likely association between uPA levels and the grade of dysplasia in colorectal adenomas have reported contradictory results (de Bruin et al, 1988; Sim et al, 1988; Suzumiya et al, 1988; Sier et al, 1991*b*). Moreover, the epithelial morphology may vary considerably among regions of individual adenomas. A microheterogeneity within adenomas might be a source of variations in previous works aiming at correlating the biochemical findings in adenoma tissue extracts with histological results in adjacent adenoma fragments (de Bruin et al, 1988; Sim et al, 1988; Suzumiya et al, 1988).

In the present study, we have correlated the grade of epithelial cell dysplasia with the extent of plasminogen activation, as well as the tPA, uPA and PAI-1 levels, in a series of large colorectal adenomas. To examine the relation between molecular changes and morphology, we have adapted the technique of histozymography to a semiquantitative assessment of plasmin generation in tissue sections. In parallel, we have performed alternating histological and biochemical evaluations in serial tissue sections. Our aim was to evaluate in situ the interplay between the histopathological changes found in the epithelium and the alterations in the plasminogen activator/plasmin system.

### MATERIALS AND METHODS

### Patients and tissue samples

Fifty-one sporadic cases of pedunculated colorectal adenomas from 19 women and 32 men (mean age 64.1 years) without a history of hereditary non-polyposis colorectal cancer, familial colorectal cancer or familial adenomatous polyposis were prospectively

*Received 10 March 1997*
*Revised 10 June 1997*
*Accepted 25 June 1997*

*Correspondence to:* G Dorta included in this study between January 1992 and October 1993. Four patients had undergone previous adenoma resection in the past 10 years. Two patients also had concurrent carcinoma of the colon and nineteen patients had one or several concurrent adenomas. All patients are currently undergoing an endoscopic and clinical follow-up. This follow-up is performed 5 years from inclusion to our study.

Adenomas were resected during elective colonoscopy and were of at least 1 cm diameter and, in cases of multiple adenomas, only the adenoma with the most advanced degree of dysplasia was included in the study. Immediately after endoscopic removal, a lateral part of the adenoma was frozen in liquid nitrogen-cooled 2-methylbutane and stored at – 80°C. The remaining part of the adenoma was referred for routine histological examination.

Biopsies, of distant but apparently normal colorectal mucosa (distant mucosa) were performed in all patients with adenoma. Three biopsies per site were taken from the hepatic flexure, the sigmoid and the rectum. All biopsies were made at least 20 cm distant from the adenoma, and the three biopsies from each location were taken within an area of 1 cm². In addition, control biopsies of normal colorectal mucosa (control mucosa) were performed in the same manner on five control patients without colorectal neoplasia or inflammatory bowel diseases; these patients underwent colonoscopy for gastrointestinal bleeding (three patients) or irritable bowel syndrome (two patients). All of these patients had an endoscopically normal colorectal mucosa. All biopsies were taken with the same type of forceps and with a standardized technique to obtain approximately identical sampling volumes. Two biopsies from each site were frozen and one was referred for routine histological examination.

The study protocol was approved by the Ethics Committee of the Faculty of Medicine of the University of Lausanne. All patients gave their written informed consent.

**Figure 1** Time of overlysis in mucosa from control subjects, in mucosa from patients with adenomas and in adenomas with low- and high-grade dysplasia. Results are presented as individual data points. Bars indicate medians; NS, not significant; *P*, *P*-values calculated using Wilcoxon's rank sum test

### Histopathological evaluations

On the lateral parts of the 51 adenomas, we made cryostat sections for biochemical and histological experiments. Cryostat tissue sections adjacent to those used for in situ zymography and antigen/activity assays were stained with haematoxylin and eosin (H&E) and examined by two experienced pathologists working in parallel. Histological type and grade of dysplasia in the adenoma were determined according to the criteria of the World Health Organization (Jass and Sobin, 1989) and of the National Polyp Study Group (Winawer et al, 1992). Low-grade dysplasia corresponds to mild and moderate dysplasia and high-grade to severe dysplasia. Adenomas were classified according to the highest grade of dysplasia in the examined section. The biopsies from normal and distant colorectal mucosa were referred for routine histopathological examination to exclude inflammatory and neoplastic lesions, e.g. aberrant crypts or microadenomas.

Because of the small amount of tissue, 2 of the 51 sets of biopsies from distant mucosa could not be studied by histozymography nor could the determinations for tPA, uPA and PAI-1 antigen concentrations and tPA activity be performed. For the same reason, three adenomas could not be analysed for tPA, uPA and PAI-1 antigen concentrations and tPA activity.

### Semiquantitative histozymography

Serial 7-μm cryostat sections of adenomas and biopsies of colorectal mucosa from hepatic flexure of patients with adenomas and controls were performed for the in situ zymographic assays for the total plasminogen activator activity (Sappino et al, 1991*a* and *b*). Sections were overlaid with 100 μl of a suspension containing 2% non-fat dry milk, 0.9% agar and 40 μg ml$^{-1}$ of purified human plasminogen (Chromogenix, Mölndal, Sweden) in phosphate-buffered saline (with 0.9 mM calcium chloride and 1 mM magnesium chloride). The overlay was covered with a glass cover slide held on standard spacers. For the determination of in situ tPA and uPA activity, the same protocol was performed applying an overlay mixture containing 1 mM amiloride, a specific uPA inhibitor (Vassalli et al, 1987), and polyclonal goat anti-human tPA immunoglobulins G (0.2 mg ml$^{-1}$) (Biopool, Umeå, Sweden) respectively. Overlay prepared in the absence of plasminogen was used as a control for non-specific caseinolytic activity. The overlaid tissue sections were incubated in a humid chamber at 37°C. The extent of caseinolysis, represented by a change to black of the overlying suspension, was followed under the microscope with dark-field illumination, and the progression of the caseinolysis was photographically documented in intervals of 30 min. The experimental end point was defined as complete caseinolysis throughout the entire area of the tissue sections. The time between the beginning and the end of the observation was called the time of overlysis (TO). uPA activity was semiquantitatively assessed with a score between 0 and 4 at 2 h from the beginning of the in situ zymographic experiment (0, no activity; 1, traces of activity; 2, low activity; 3, moderate activity; and 4, high activity).

### Tissue extraction and protein concentration

Tissue extracts of adenomas were prepared from consecutive 250 μm-cryostat sections. Tissue extracts from colorectal mucosa, distant and control, were obtained from pooled biopsies (one from the hepatic flexure, two from the sigmoid and two from the

© Cancer Research Campaign 1998

**Figure 2** In situ zymographies after 90 min of incubation in two consecutive sections of distant mucosa (1A and 1B), of an adenoma with low-grade dysplasia (2A and 2B) and of an adenoma with high-grade dysplasia (3A and 3B). In the A series, tPA activity is blocked with anti-tPA antibodies and, in the B series, uPA activity is blocked by amiloride. Plasminogen-mediated caseinolysis appears as dark zones on the tissue sections. Plasminogen-mediated caseinolysis is tPA mediated in the mucosa specimen (1A negative, 1B extensive caseinolysis). Adenomas show both tPA- and uPA-mediated caseinolysis (2A and 2B, 3A and 3B). uPA-mediated activity is located in the periphery of the adenoma sections (2A and 3A), while tPA activity is distributed diffusely over the tissue sections. Bar = 1 mm

**Figure 3** Concentration of tPA antigen levels (**A**) and tPA activity levels (**B**) in control subjects, in mucosa from patients with adenomas and in adenomas with low- and high-grade dysplasia. Results are presented as individual data points. Bars indicate medians; NS, not significant; *P*, *P*-value of Wilcoxon's rank sum test rectum). The samples were homogenized by ultrasound sonication in 200–300 µl of Camiolo buffer (0.075M potassium acetate, 0.3 M natrium chloride, 0.1 M arginine, 0.01 M EDTA) with 0.25% Triton X-100, pH 4.2, at 4°C as described elsewhere (Markus et al, 1983). The homogenates were centrifuged at 12 000 *g* for 10 min at 4°C. The protein concentration in the supernatant was assessed using the dye-binding Bio-Rad protein assay (Bradford, 1976) (Bio-Rad, Richmond, CA, USA).

### Determination of PAI-1, uPA and tPA antigen levels and tPA activity

The total amount of PAI-1 antigen, i.e. latent, active and in complex with activators, was determined by TintElize PAI-1 (Biopool, Umeå, Sweden). To increase the PAI-1 detection limit to 0.3 ng ml$^{-1}$, sample volumes of 40 µl were used instead of the recommended 20-µl volume (Sier et al, 1991*a*). The total amount of uPA antigen was determined by TintElize uPA (Biopool). t-PA antigen concentration was determined by TintElize tPA (Biopool). The activity of tPA in tissue extracts was determined by Chromolize tPA (Biopool).

Because the sample volumes from adenomas and biopsies were very small, the amount of tissue available for the assays was correspondingly low. Therefore, only tPA, uPA and PAI-1 antigen levels and tPA activity could be analysed.

### Calculations and statistics

Antigen concentrations for uPA, tPA and PAI-1 are expressed as ng of antigen per mg of total protein. tPA activities are expressed as international units per mg of total protein. Times of overlysis (TO) are expressed in h. Results are given as mean ± s.e.m. In the figures, medians or medians with quantiles are used for description of both parametric and non-parametric data. Differences between group means were tested for significance using the Wilcoxon's rank sums test. Differences in scores were tested using the median test. When both the factor and the response were nominal, the chi-square test was performed. Correlations were tested using linear regression analysis. Significance was defined as $P < 0.05$.

### RESULTS

### Size of resected adenomas and histological evaluation

Of the 51 adenomas, 12 were 1 cm, 23 were 1.1–2 cm and 16 adenomas were more than 2 cm in diameter, as measured before fixation.

Histologically, there were 28 tubular, 19 tubulovillous and four villous adenomas. Thirty-one adenomas were classified as being low-grade and 20 as being high-grade dysplastic.

Nine of 28 tubular adenomas as well as 8 out of 19 tubulovillous adenomas showed high-grade dysplasia ($P = 0.49$ high-grade dysplasia in tubular vs high-grade dysplasia in tubulovillous). All four villous adenomas showed high-grade dysplasia. Villous adenomas were more likely to be high-grade dysplastic than those of tubular and tubulovillous architecture ($P < 0.04$).

No correlation was observed between the TO, the uPA, PAI-1 and tPA levels or the size and the histological type of adenomas, categorized as tubular, villous or tubulovillous.

In addition to the histopathological evaluation of adenomas, biopsy samples of distant colorectal mucosa from adenomas as well as those from controls were analysed. None of these showed any adenomatous or inflammatory changes.

### Histozymography

Figure 1 shows the values of TO, as defined in the Materials and methods section. TO was not different in five samples of control

© Cancer Research Campaign 1998

**Figure 4** Concentration of uPA antigen measured by enzyme-linked immunosorbent assay in control subjects, in mucosa from patients with adenomas and in adenomas with low- and high-grade dysplasia. Results are presented as individual data points. Bars indicate medians; NS, not significant; *P*, *P*-values calculated using Wilcoxon's rank sum test mucosa (1.50 ± 0.08 h, mean ± s.e.m.) compared with 49 samples of distant mucosa taken from the adenoma patients (1.51 ± 0.05 h, $P = 0.80$). In contrast, the mean of the TO values in both control and distant mucosae is markedly lower than that of 51 adenomas (3.79 ± 0.22 h, $P < 0.001$), indicating that the level of plasminogen activation was diminished in adenomas compared with mucosae. There was no significant difference in the TO between the 31 adenomas with low-grade dysplasia (3.45 ± 0.16 h) and the 20 adenomas with high-grade dysplasia (4.33 ± 0.46 h, $P = 0.26$).

Figure 2 illustrates the expression of in situ caseinolytic activities assessed in the presence of anti-tPA antibodies (Figure 2 1A–3A) or amiloride (Figure 2 1B–3B) in two consecutive sections of the same sample of distant mucosa (Figure 2 1A and 1B), adenoma with low-grade dysplasia (Figure 2 2A and 2B) and adenoma with high-grade dysplasia (Figure 2 3A and 3B). The histozymograms of normal mucosae show that lysis was inhibited by anti-tPA antibodies (Figure 2 1A) but not by amiloride (Figure 2 1B), indicating that it was tPA mediated. In adenomas, tPA-mediated caseinolysis appeared to be associated with central regions (Figure 2, 2B and 3B), whereas uPA-mediated lysis was predominant in the periphery (Figure 2, 2A and 3A).

No uPA activity was detected in five sample sets of control mucosa. uPA activity was present in 11 out of 49 distant mucosae sample sets ($P = 0.12$, chi-square test, controls vs distant). All adenomas were positive for uPA activity ($P < 0.001$, chi-square test, control mucosae vs adenomas; $P < 0.001$, distant mucosae vs adenomas). tPA activity was present in all mucosa and adenoma samples.

### tPA antigen concentration

Figure 3A shows tPA antigen concentrations in five samples of control mucosa and 49 samples of distant mucosae, and in 48 adenomas with low- and high-grade dysplasia. There was no significant difference in tPA antigen concentrations between control mucosae (58.95 ± 5.95 ng mg$^{-1}$ protein) and distant mucosae (49.46 ± 2.39 ng mg$^{-1}$ protein, $P = 0.19$). Both control and distant mucosae had higher tPA antigen concentrations than adenomas (7.88 ± 0.47 ng mg$^{-1}$ protein, $P < 0.001$). Moreover, the tPA antigen levels in normal and distant mucosae showed no overlap with those in adenomas. The tPA antigen concentration in 29 adenomas with low-grade dysplasia (8.01 ± 0.60 ng mg$^{-1}$ protein) was not different from that in 19 adenomas with high-grade dysplasia (7.69 ± 0.76 ng mg$^{-1}$ protein, $P = 0.95$).

### tPA activity

The tPA activity levels in control and distant mucosae and in adenomas with low- and high-grade dysplasia are reported in Figure 3B. The tPA activity levels in control mucosa (21.47 ± 3.20 IU mg$^{-1}$ protein) did not differ from those in distant mucosa (19.44 ± 1.20 IU mg$^{-1}$ protein, $P = 0.53$). Both the control and the distant mucosae had extremely higher levels of tPA activity than did adenomas (3.71 ± 0.20 IU mg$^{-1}$ protein, $P < 0.001$). There was no significant difference in tPA activity levels between adenomas with low-grade (3.92 ± 0.26 IU mg$^{-1}$ protein) and those with high-grade (3.38 ± 0.28 IU mg$^{-1}$ protein, $P = 0.18$) dysplasia.

In addition, there was a linear correlation between tPA antigen and activity levels both in distant mucosa ($r = 0.88$, $P < 0.001$) and in adenomas ($r = 0.84$, $P < 0.001$; data not shown).

### PAI-1 concentration

In 33 of 49 samples of distant mucosa and in control mucosa, PAI-1 levels were below the detection limit. In all these samples, PAI-1 antigen concentrations were considered as being equal to the detection threshold. PAI-1 concentrations in control mucosae (0.11 ± 0.02 ng mg$^{-1}$ protein) were not significantly different from those in distant mucosae (0.17 ± 0.01 ng mg$^{-1}$ protein, $P = 0.08$). In contrast, in 48 adenomas, the levels of PAI-1 were higher (0.93 ± 0.14 ng mg$^{-1}$ protein) than those in control and distant mucosae ($P < 0.001$). PAI-1 antigen concentrations in adenomas with high-grade dysplasia (1.20 ± 0.31 ng mg$^{-1}$ protein) were not different from those in adenomas with low-grade dysplasia (0.76 ± 0.11 ng mg$^{-1}$ protein, $P = 0.78$).

In addition, PAI-1 concentration was negatively correlated with tPA concentration ($r = -0.36$, $P = 0.01$) and tPA activity ($r = -0.44$, $P < 0.002$; data not shown).

### uPA concentration

Figure 4 shows the uPA antigen concentrations in control and distant mucosae as well as in adenomas with low- and high-grade dysplasia. No significant difference in uPA antigen concentration was found between five control mucosae (1.31 ± 0.13 ng mg$^{-1}$ protein) and 49 distant mucosae (1.48 ± 0.14 ng mg$^{-1}$ protein, $P =$ 0.86). The 48 adenomas had higher uPA antigen concentrations (4.38 ± 0.31 ng mg$^{-1}$ protein) than control ($P = 0.001$) and distant mucosae ($P < 0.001$). The adenomas with low-grade dysplasia had lower uPA antigen concentrations (3.66 ± 0.31 ng mg$^{-1}$ protein) than did adenomas with high-grade dysplasia (5.48 ± 0.56 ng mg$^{-1}$ protein, $P < 0.01$), indicating that uPA protein concentration correlates with the degree of dysplasia in colorectal adenomatous epithelium.

© Cancer Research Campaign 1998

In addition, there was a positive linear correlation between uPA and PAI-1 antigen concentrations in adenomas ($r = 0.73$, $P < 0.001$; data not shown).

### uPA activity

The uPA-mediated plasminogen-dependent caseinolysis was scored on tissue sections by histozymography as defined in the Material and methods. The adenomas had distinctly higher scores for caseinolysis (2.30 ± 0.10) than did controls (0.00 ± 0.00) or distant mucosae (0.29 ± 0.09, $P < 0.001$), indicating that uPA activity is increased in adenomas. Moreover, the adenomas with high-grade dysplasia showed higher scores for caseinolysis (2.57 ± 0.19) than those with low-grade dysplasia (2.12 ± 0.09, $P < 0.03$). There was no difference between the controls and distant mucosae ($P = 0.25$).

### uPA/PAI-1 ratio and the grade of dysplasia

The uPA/PAI-1 antigen ratios were calculated in 48 adenomas. There was a trend for lower uPA/PAI-1 ratios in 29 adenomas with low-grade dysplasia (6.83 ± 1.25) than in 19 adenomas with high-grade dysplasia (11.55 ± 2.06, $P = 0.09$).

### DISCUSSION

In the present study, we performed a combination of quantitative antigen/activity determinations, semiquantitative histozymographic analyses and histopathological assessments in consecutive cryostat tissue sections of adenomas and normal colorectal mucosa. We have been able to correlate the alterations in the plasminogen activator/plasmin proteolytic system found in colorectal adenomas with the grade of epithelial cell dysplasia. Using semiquantitative histozymography, we have shown that plasminogen activator-mediated caseinolytic activity is diminished in adenomas compared with mucosa samples. In normal mucosa, the major source of plasminogen-mediated caseinolysis is due to tPA, hence the decreased level of plasminogen activation in adenomas can be assigned to lowered tPA activity. These histozymographic data on tPA are in agreement with the results of previous studies on tissue homogenates (de Bruin et al, 1988; Suzumiya et al, 1988; Sier et al, 1991*a*). Using antigen/activity immunosorbent methods together with an efficient tissue extraction procedure (Camiolo et al, 1982), we could demonstrate a clear-cut reduction in the tPA levels in adenomas compared with control and distant mucosae. The tPA antigen assay used in this study detects both free tPA and tPA complexed with inhibitor (Ranby et al, 1989). Additionally, we showed a linear correlation between the values of tPA antigen and activity in both mucosae and adenomas. Thus, low tPA activity in adenomas can mainly be attributed to a low protein level rather than to an effect of inhibitors alone. This observation contradicts the previous hypothesis claiming that diminution of tPA catalytic activity is linked to the up-regulation of endothelial PAI-1 in the stromal compartment (Pyke et al, 1991*a*; Delbaldo et al, 1995). tPA is mainly involved in intravascular fibrinolysis and in both normal and neoplastic colorectum. tPA mRNA is readily detected in the endothelium by in situ hybridization (Pyke et al, 1991*a*, Delbaldo et al, 1995). Human neoplastic cells are known to constitutively produce a variety of growth factors, cytokines and positive chemotactic substances (Herlyn et al, 1991), and some of these mediators, such as tumour necrosis factor $\alpha$ and interleukin-1$\beta$, have been shown to down-regulate the tPA expression in human endothelial cells (Bevilacqua et al, 1986; Schleef et al, 1988), suggesting that dysplastic colorectal epithelium may down-regulate the tPA expression in the stroma by a paracrine mechanism. Another explanation is that the minor effect of inhibitors on tPA activity found in adenomas results from the direct degradation of tPA/PAI-1 complexes in tissue by monocytes, as recently suggested using an in vitro model (Simon et al, 1995). The lack of significant differences in tPA levels between adenomas with different grades of dysplasia suggests that a major decrease in tPA levels occurs at an early stage of adenoma formation. In colorectal cancer patients, low tPA levels in apparently normal colorectal mucosa adjacent to tumour are associated with a poor overall survival (Ganesh et al, 1994). This finding, together with our observations, suggests that the role of tPA in the adenoma–carcinoma sequence is perhaps more important than postulated in previous studies. In our opinion, further studies examining the mechanism of the down-regulation of tPA are needed to bring insight onto the early stages of colorectal carcinogenesis.

Antigen levels of PAI-1 were higher in adenomas and in distant mucosa of subjects with an adenoma than in mucosa samples from normal subjects. Similar to tPA, no correlation between the grade of epithelial dysplasia and the level of PAI-1 expression in adenomas was found. This result is consistent with our previous report that tissue distribution of PAI-1 mRNA does not correlate with the grade of dysplasia (Sordat et al, 1997). We thus assume that PAI-1 up-regulation is an early event associated with adenoma formation. Our data, and those of others, have demonstrated that endothelial cells of colorectal adenomas as well as of carcinomas can accumulate PAI-1 mRNA (Pyke et al, 1991*a*; Delbaldo et al, 1995; Sordat et al, 1997). Experimentally, PAI-1 is expressed by migrating endothelial cells (Pepper et al, 1992). It has been shown in vitro that stimulation of angiogenesis results in an increase in plasminogen activator activity followed by a rise in PAI-1 expression to limit the excessive plasmin generation (Flaumenhaft et al, 1992). Therefore, we attribute this up-regulation of PAI-1 to paracrine modulations in the adenomatous stroma associated with neo-angiogenesis and extracellular matrix remodelling.

As this study was based on endoscopically removed tissue samples, the material was not large enough to perform PAI-2 determinations. However, other authors have demonstrated that PAI-2 levels increase in parallel with PAI-1 levels (Sier et al, 1991*a*).

Using histozymography and antigen determination, we have shown that uPA antigen as well as uPA-mediated caseinolysis is increased in adenomas compared with control and distant mucosae, this agrees with results from previous studies on tissue homogenates (de Bruin et al, 1988; Suzumiya et al, 1988; Sier et al, 1991*a*). Interestingly, in about 20% of patients with adenomas, uPA-mediated caseinolysis was found in sections of distant mucosa but was not detected in any of the control mucosae. It has been reported previously that mucosae adjacent to colorectal cancer express uPA activity levels similar to those from healthy subjects (Gibson et al, 1991). However, other studies have shown that histologically normal colorectal mucosa from cancer patients had a higher proliferation activity than its counterpart from normal subjects (Terpstra et al, 1987; Ponz de Leon et al, 1988). Therefore, we hypothesize that the proteolytic profile in adenoma-bearing mucosa is, at least in some instances, different from the mucosa from control subjects without colorectal neoplasia.

We have demonstrated that both uPA antigen and uPA-mediated caseinolysis were highest in the adenomas with high-grade

© Cancer Research Campaign 1998

dysplasia. This result is consistent with our recent report that stromal expression of uPA mRNA in adenomas correlates with the grade of dysplasia in adjacent epithelium (Sordat et al, 1997). In previous studies, a correlation between uPA levels and the grade of dysplasia could either not be established (de Bruin et al, 1988; Sim et al, 1988) or it was suggested using a small series of adenomas (Suzumiya et al, 1988). A combined determination of uPA levels and dysplasia grade in consecutive cryostat sections allowed us to demonstrate the significant correlation between biochemical events and histopathological findings in adenomas. Several studies showed that in both colorectal cancer and adenoma, the changes in uPA levels originate in the stroma (Pyke et al, 1991*b*; Koretz et al, 1993; Delbaldo et al, 1995; Sordat et al, 1997). Thus, the parallel increase in uPA and PAI-1 antigen levels detected in adenomas suggests that both components might be regulated, at least in part, by the same paracrine stimuli. However, only the uPA levels correlated with the grade of dysplasia, possibly shifting the protease–antiprotease balance in favour of higher net proteolytic activity in the areas of high-grade dysplasia. Indeed, there was a trend for lower uPA/PAI-1 antigen ratios in low-grade dysplastic adenomas compared with high-grade dysplastic adenomas. As high uPA levels associate with metastatic potential in various types of human cancers (Foekens et al, 1992; Schmitt et al, 1992; Sumiyoshi et al, 1992; Grondahl-Hansen et al, 1993; Pujade-Lauraine et al, 1993), we hypothesize that this protease–antiprotease imbalance found in high-grade dysplasia represents a marker for the transition towards the invasive behaviour in adenomatous lesions.

In conclusion, we have shown that the grade of epithelial dysplasia in the colorectum correlates with defined alterations in the plasminogen activator/plasmin system. These observations support the hypothesis that high-grade dysplasia is a critical marker of malignant transition in colorectal adenoma. The clinical relevance of these findings will be established in our future studies, after completed follow-up of all patients.

### ABBREVIATIONS

PAI-1, plasminogen activator inhibitor type 1; PAI-2, plasminogen activator inhibitor type 2; TO, time of overlysis; tPA, tissue-type plasminogen activator; uPA, urokinase-type plasminogen activator

### ACKNOWLEDGEMENTS

This study was supported by grants from Cancer Research Switzerland (AKT 328, 334 and 589), the Swiss National Science Foundation (nos. 31-33993.92 and 31-04500.95), the Neuchâtel Cancer League, the Charles Veillon and the Emma Muschamp Foundations, Lausanne, Switzerland.

### REFERENCES

Bevilacqua MP, Schleef RR, Gimbrone MA and Loskutoff DJ (1986) Regulation of the fibrinolytic system of cultured human vascular endothelium by interleukin 1. *J Clin Invest* **78**: 587–591

Blasi F (1993) Molecular mechanisms of protease-mediated tumor invasiveness. *J Surg Oncol Suppl* **3**: 21–23

Bradford MM (1976) A rapid and sensitive method for the quantitation of microgram quantities of protein utilizing the principle of protein-dye binding. *Anal Biochem* **72**: 248–254

Camiolo SM, Siuta MR and Madeja JM (1982) Improved medium for extraction of plasminogen activator from tissue. *Prep Biochem* **12**: 297–305

De Bruin PAF, Griffioen G, Verspaget HW, Verheijen JH and Lamers CBHW (1987) Plasminogen activators and tumor development in the human colon: activity levels in normal mucosa, adenomatous polyps, and adenocarcinomas. *Cancer Res* **47**: 4654–4657

De Bruin PA, Griffioen G, Verspaget HW, Verheijen JH, Dooijewaard G, Van Den Ingh HF and Lamers CB (1988) Plasminogen activator profiles in neoplastic tissues of the human colon. *Cancer Res* **48**: 4520–4524

Delbaldo C, Cunningham M, Vassalli JD and Sappino AP (1995) Plasmin-catalyzed proteolysis in colorectal neoplasia. *Cancer Res* **55**: 4688–4695

Flaumenhaft R, Abe M, Mignatti P and Rifkin DB (1992) Basic fibroblast growth factor-induced activation of latent transforming growth factor beta in endothelial cells: regulation of plasminogen activator activity. *J Cell Biol* **118**: 901–909

Foekens JA, Schmitt M, Van Putten WL, Peters HA, Bontebal M, Janicke F and Klijn JG (1992) Prognostic value of urokinase-type plasminogen activator in 671 primary breast cancer patients. *Cancer Res* **52**: 6101–6105

Ganesh S, Sier CF, Griffioen G, Vloedgraven HJ, De Boer A, Welwaart K, Van De Velde CJ, Van Krieken JH, Verheijen JH, Lamers CB and Verspaget HW (1994) Prognostic relevance of plasminogen activators and their inhibitors in colorectal cancer. *Cancer Res* **54**: 4065–4071

Gelister JS, Mahmoud M, Lewin MR, Gaffney PJ and Boulos PB (1986) Plasminogen activators in human colorectal neoplasia. *Br Med J* **293**: 728–731

Gibson PR, Van De Pol E and Doe WF (1991) Cell associated urokinase activity and colonic epithelial cells in health and disease. *Gut* **32**: 191–195

Grondahl-Hansen J, Christensen IJ, Rosenquist C, Brünner N, Mouridsen HT, Dano K and Blichert-Toft M (1993) High levels of urokinase-type plasminogen activator and its inhibitor PAI-1 in cytosolic extracts of breast carcinomas are associated with poor prognosis. *Cancer Res* **53**: 2513–2521

Hamilton SR (1992) The adenoma–adenocarcinoma sequence in the large bowel: variations on a theme. *J Cell Biochem Suppl* **16G**: 41–46

Herlyn M and Malkowicz SB (1991) Regulatory pathways in tumor growth and invasion. *Lab Invest* **65**: 262–271

Jass JR and Sobin LH (1989) Histological typing of intestinal tumors. In *WHO International Histological Classification of Tumors*, pp. 29–30. Springer: Berlin

Konishi F and Morson BC (1982) Pathology of colorectal adenomas: a coloscopic survey. *J Clin Pathol* **35**: 830–841

Koretz K, Moller P and Schwartz-Albiez R (1993) Plasminogen activators and plasminogen activator inhibitors in human colorectal carcinoma tissues are not expressed by the tumour cells. *Eur J Cancer* **29A**: 1184–1189

Markus G, Camiolo SM, Kohga S, Madeja JM and Mittelman A (1983) Plasminogen activator secretion of human tumors in short-term organ culture, including a comparison of primary and metastatic colon tumors. *Cancer Res* **43**: 5517–5525

Muto T, Bussey HJ and Morson BC (1975) The evolution of cancer of the colon and rectum. *Cancer* **36**: 2251–2270

O'Brien MJ, Winawer SJ, Zauber AG, Gottlieb LS, Diaz B, Dickersin GR, Ewing S, Geller S, Kasimian D and the National Polyp Study Workgroup (1990) The National Polyp Study. Patient and polyp characteristics associated with high-grade dysplasia in colorectal adenomas. *Gastroenterology* **98**: 371–379

Pepper MS, Sappino AP, Montesano R, Orci L and Vassalli JD (1992) Plasminogen activator inhibitor-1 is induced in migrating endothelial cells. *J Cell Physiol* **153**: 129–139

Ponz De Leon M, Roncucci L, Di Donato P, Tassi L, Smerieri O, Amorico MG, Malagoli G, De Maria D, Antonioli A, Chahin NJ, Perini M, Rigo G, Barberini G, Manenti A, Biasco G and Barbara L (1988) Pattern of epithelial cell proliferation in colorectal mucosa of normal subjects and of patients with adenomatous polyps or cancer of the large bowel. *Cancer Res* **48**: 4121–4126

Pujade-Lauraine E, Lu H, Mirshahi S, Soria J, Soria C, Bernadou A, Kruithof EK, Lijnen HR and Burtin P (1993) The plasminogen-activation system in ovarian tumors. *Int J Cancer* **55**: 27–31

Pyke C, Kristensen P, Ralfkiaer E, Eriksen J and Dano K (1991*a*) The plasminogen activation system in human colon cancer: messenger RNA for the inhibitor PAI-1 is located in endothelial cells in the tumor stroma. *Cancer Res* **51**: 4067–4071

Pyke C, Kristensen P, Ralfkiaer E, Grondahl-Hansen J, Eriksen J, Blasi F and Dano K (1991*b*) Urokinase-type plasminogen activator is expressed in stromal cells and its receptor in cancer cells at invasive foci in human colon adenocarcinomas. *Am J Pathol* **138**: 1059–1067

Ranby M, Nguyen G, Scarabin PY and Samama M (1989) Immunoreactivity of tissue plasminogen activator and of its inhibitor complexes: biochemical and multicenter validation of a two site immunosorbent assay. *Thromb Haemost* **61**: 409–414

© Cancer Research Campaign 1998

Sappino AP, Belin D, Huarte J, Hirschel-Scholz S, Saurat JH and Vassalli JD (1991*a*) Differential protease expression by cutaneous squamous and basal cell carcinomas. *J Clin Invest* **88**: 1073–1079

Sappino AP, Huarte J, Vassalli JD and Belin D (1991*b*) Sites of synthesis of urokinase and tissue-type plasminogen activators in the murine kidney. *J Clin Invest* **87**: 962–970

Schleef RR, Bevilacqua MP, Sawdey M, Gimbrone MA Jr and Loskutoff DJ (1988) Cytokine activation of vascular endothelium. Effects on tissue-type plasminogen activator and type 1 plasminogen activator inhibitor. *J Biol Chem* **263**: 5797–5803

Schmitt M, Jänicke F, Moniwa N, Chucholowski N, Pache L and Graeff H (1992) Tumor-associated urokinase-type plasminogen activator: biological and clinical significance. *Biol Chem Hoppe Seyler* **373**: 611–622

Sier CFM, Verspaget HW, Griffioen G, Verheijen JH, Quax PHA, Dooijewaard G, De Bruin PAF and Lamers CB (1991*a*) Imbalance of plasminogen activators and their inhibitors in human colorectal neoplasia. Implications of urokinase in colorectal carcinogenesis. *Gastroenterol* **101**: 1522–1528

Sier CFM, Fellbaum C, Verspaget HW, Schmitt M, Griffioen G, Graeff H, Höfler H and Lamers CB (1991*b*) Immunolocalization of urokinase-type plasminogen activator in adenomas and carcinomas of the colorectum. *Histopathology* **19**: 231–237

Sim PS, Stephens RW, Fayle DR and Doe WF (1988) Urokinase-type plasminogen activator in colorectal carcinomas and adenomatous polyps: quantitative expression of active and proenzyme. *Int J Cancer* **42**: 483–488

Simon DI, Xu H and Vaughan DE (1995) Cathepsin D-like aspartyl protease activity mediates the degradation of tissue-type plasminogen activator/plasminogen activator inhibitor-1 complexes in human monocytes. *Biochem Biophys Acta* **1268**: 143–151

Sordat I, Chaubert P, Protiva P, Guillou L, Mazzucchelli L, Benhattar J, Tran-Thang C, Blum AL, Dorta G and Sordat B (1997) In situ stromal expression of the urokinase/plasmin system correlates with epithelial dysplasia in colorectal adenomas. *Am J Pathol* **150**: 283–295

Sumiyoshi K, Serizawa K, Urano T, Takada Y, Takada A and Baba S (1992) Plasminogen activator system in human breast cancer. *Int J Cancer* **50**: 345–348

Suzumiya J, Hasui Y, Kohga S, Sumiyoshi A, Hashida S and Ishikawa E (1988) Comparative study of plasminogen activator antigens in colonic carcinomas and adenomas. *Int J Cancer* **42**: 627–632

Terpstra OT, Van Blankenstein M, Dees J and Eilers GA (1987) Abnormal pattern of cell proliferation in the entire colonic mucosa of patients with colon adenoma or cancer. *Gastroenterology* **92**: 704–708

Vassalli JD and Belin D (1987) Amiloride selectively inhibits the urokinase-type plasminogen activator. *FEBS Lett* **214**: 187–191

Vassalli JD, Sappino AP and Belin D (1991) The plasminogen activator/plasmin system. *J Clin Invest* **88**: 1067–1072

Winawer SJ, Zauber AG, O'Brien MJ, Gottlieb LS, Sternberg SS, Stewart ET, Bond JH, Schapiro M, Panish JF, Waye JD, Kurtz RC, Shike M, May Nah Ho and the National Polyp Study Workgroup (1992) The National Polyp Study. Design, methods, and characteristics of patients with newly diagnosed polyps. The National Polyp Study Workgroup. *Cancer Suppl* **70**: 1236–1245

© Cancer Research Campaign 1998